# Genistein–Butein Co-Treatment Suppresses Glycolytic Metabolism and Induces Apoptotic Signaling in PC-3 Prostate Cancer Cells

**DOI:** 10.3390/cimb48030258

**Published:** 2026-02-27

**Authors:** Moon-Kyun Cho, Yeji Lee, Sang-Han Lee, Hae-Seon Nam, Yoon-Jin Lee

**Affiliations:** 1 Division of Molecular Cancer Research, Soonchunhyang Medical Research Institute, Soonchunhyang University, Cheonan 31511, Republic of Korea; mkcho@schmc.ac.kr (M.-K.C.); m1037624@sch.ac.kr (S.-H.L.); namhs@sch.ac.kr (H.-S.N.); 2 Department of Dermatology, Soonchunhyang University Hospital, Seoul 04401, Republic of Korea; 3 Department of Biochemistry, College of Medicine, Soonchunhyang University, Cheonan 31511, Republic of Korea; yjyjlee37@naver.com; 4 Department of Tropical Medicine, College of Medicine, Soonchunhyang University, Cheonan 31511, Republic of Korea

**Keywords:** genistein, butein, prostate cancer, glycolytic metabolism, apoptotic signaling, combination treatment, PC-3 cells

## Abstract

Prostate cancer progression involves metabolic reprogramming that supports sustained proliferation and survival, highlighting metabolic pathways as potential targets for intervention. While genistein (GEN) and butein (BTN) are naturally occurring polyphenolic compounds with reported anticancer activities, their combined effects on prostate cancer cell metabolism and apoptotic signaling remain unclear. Here, we investigated the effects of GEN and BTN, administered individually and in combination, on human PC-3 prostate cancer cells, with normal human prostate epithelial cells (HPrEC) used for comparison. Cell viability was assessed using MTT and trypan blue exclusion assays. Glycolytic metabolism was evaluated by measuring glucose consumption, lactate production, hexokinase and pyruvate dehydrogenase activity, and intracellular ATP levels, while apoptotic and survival signaling pathways were analyzed by means of Annexin V staining and Western blotting. GEN/BTN co-treatment selectively reduced PC-3 cell viability, producing greater inhibitory effects than either compound alone. This enhanced response was accompanied by suppression of glycolytic metabolism, ATP depletion, attenuation of AKT and ERK phosphorylation, and activation of apoptotic signaling, as evidenced by increased cleavage of caspase-3 and PARP. Collectively, these findings indicate that GEN/BTN co-treatment cooperatively disrupts glycolytic metabolism while activating apoptotic signaling in prostate cancer cells.

## 1. Introduction

Despite advances in early detection and treatment strategies, prostate cancer—one of the most frequently diagnosed malignancies among men worldwide—remains a leading cause of cancer-related mortality [1,2]. Although localized disease can often be managed effectively, disease progression and resistance to conventional therapies result in advanced and androgen-independent prostate cancer continuing to present significant therapeutic challenges [2]. These limitations have prompted growing interest in alternative strategies that target fundamental cellular processes sustaining tumor growth and survival.

Metabolic reprogramming is now recognized as a hallmark of cancer, enabling malignant cells to meet increased energetic and biosynthetic demands while adapting to stressful microenvironments [3]. Prostate cancer cells, including androgen-independent PC-3 cells, exhibit pronounced alterations in glucose metabolism characterized by increased reliance on aerobic glycolysis, even in the presence of sufficient oxygen [4]. This metabolic shift supports rapid proliferation and contributes to aggressive tumor behavior, making glycolytic pathways attractive targets for anticancer intervention [5].

Key enzymes involved in glucose metabolism play critical roles in regulating glycolytic flux and cellular energy balance. Hexokinase (HK), particularly the HK2 isoform, catalyzes the first committed step of glycolysis and is frequently upregulated in cancer cells, thereby enhancing glucose consumption and lactate production [6]. In parallel, pyruvate dehydrogenase (PDH) regulates the conversion of pyruvate to acetyl–CoA, linking glycolysis to mitochondrial oxidative metabolism. Dysregulation or reduced PDH activity limits pyruvate entry into the tricarboxylic acid cycle, reinforcing dependence on glycolysis for cellular bioenergetic demands [7]. Together, increased HK activity and impaired PDH function contribute to metabolic flexibility that supports cancer cell survival [4,5].

In addition to metabolic alterations, aberrant activation of pro-survival signaling pathways is a defining feature of prostate cancer progression. In particular, the phosphoinositide 3-kinase (PI3K)/AKT and extracellular signal-regulated kinase (ERK) pathways are frequently activated and promote cell growth, metabolic adaptation, and resistance to apoptosis [8,9]. Phosphorylation of AKT and ERK enhances glycolytic activity and suppresses apoptotic signaling, thereby coupling metabolic regulation with survival pathways. Conversely, inhibition of these signaling cascades can trigger apoptotic responses characterized by caspase activation and cleavage of downstream substrates, such as poly(ADP-ribose) polymerase (PARP) [10]. This close interplay between metabolism and survival signaling highlights the potential benefit of strategies that concurrently disrupt metabolic homeostasis and activate apoptotic pathways.

Owing to their diverse biological activities and generally favorable safety profiles, naturally occurring dietary polyphenols have gained increasing attention as potential anticancer agents [11]. Genistein (GEN), a soy-derived isoflavone, has been reported to exert antiproliferative and pro-apoptotic effects in various cancer models, including prostate cancer [11,12]. Mechanistic studies suggest that GEN modulates multiple pathways involved in cell cycle regulation, apoptosis, and metabolism [12]. Butein (BTN), a chalcone-type polyphenol isolated from several medicinal plants, has also demonstrated anticancer activity through inhibition of tumor cell growth and induction of apoptotic signaling in different cancer types [13].

Despite evidence supporting the individual anticancer properties of GEN and BTN, their combined effects on prostate cancer cells remain insufficiently characterized, particularly with respect to metabolic regulation. Combination strategies employing bioactive phytochemicals may enhance biological efficacy by simultaneously targeting multiple cellular processes while potentially limiting toxicity associated with higher doses of single agents [14]. However, careful evaluation is required to determine whether combined treatment produces effects beyond those observed with individual compounds.

An additional consideration in the development of anticancer strategies is selectivity toward malignant cells. Normal prostate epithelial cells exhibit distinct metabolic and signaling characteristics compared with prostate cancer cells, raising the possibility that cancer-specific metabolic vulnerabilities may be selectively exploited [15]. Comparative analyses between malignant and non-malignant prostate cells therefore provide important insight into the specificity and translational relevance of candidate approaches.

Based on these considerations, the present study examined the effects of genistein (GEN) and butein (BTN), administered individually and in combination, on glycolytic metabolism and apoptotic signaling in human PC-3 prostate cancer cells, with normal human prostate epithelial cells used for comparison. Glucose consumption, lactate production, hexokinase and PDH activity, intracellular ATP levels, and key signaling molecules associated with survival and apoptosis were assessed to determine whether GEN/BTN co-treatment disrupts metabolic homeostasis and promotes apoptotic signaling in prostate cancer cells. Elucidation of the metabolic and molecular consequences of this co-treatment may provide a rational basis for further exploration of polyphenol-based combination strategies in prostate cancer research.

## 2. Materials and Methods

### 2.1. Cell Culture

Human prostate cancer PC-3 cells and normal human prostate epithelial cells (HPrEC) were obtained from the American Type Culture Collection (ATCC; Manassas, VA, USA). PC-3 cells were maintained in Dulbecco’s Modified Eagle Medium (DMEM), whereas HPrEC cells were cultured in epithelial cell growth medium, in accordance with the supplier’s recommendations. All culture media were supplemented with 10% fetal bovine serum and 1% penicillin–streptomycin. Cells were incubated at 37 °C in a humidified atmosphere containing 5% CO_2_, and were routinely passaged at (70–80)% confluence. Only cells in the logarithmic growth phase were used for subsequent experiments.

### 2.2. Reagents and Treatment Conditions

GEN and BTN were purchased from Sigma-Aldrich (St. Louis, MO, USA). Stock solutions were prepared in dimethyl sulfoxide (DMSO) and stored at −20 °C protected from light. For all experiments, GEN and BTN were freshly diluted in culture medium, with the final concentration of DMSO not exceeding 0.1% (*v*/*v*). Cells were treated with GEN (100 μM), BTN (15 μM), or their combination (GEN/BTN) at the indicated concentrations for 24 or 48 h, while vehicle-treated cells served as controls.

### 2.3. Cell Viability Assays

Cell viability was assessed using MTT and trypan blue exclusion assays. For the MTT assay, cells were seeded in 96-well plates at a density of 1 × 10^4^ cells per well and allowed to attach overnight. Cells were then treated with GEN, BTN, or their combination at the indicated concentrations for 24 or 48 h. Following treatment, MTT solution (final concentration, 0.1 mg/mL) was added and incubated to allow formazan formation. The resulting formazan crystals were dissolved in dimethyl sulfoxide (DMSO), and absorbance was measured at 570 nm using a microplate reader (GlomMax-Multi Detection System; Promega, Madison, WI, USA).

For the trypan blue exclusion assay, cells were harvested after treatment, resuspended in phosphate-buffered saline, and stained with trypan blue solution. Viable and nonviable cells were distinguished based on membrane integrity and counted using a hemocytometer. Cell viability was expressed as the percentage of viable cells relative to untreated control cells, which were defined as 100%. All experiments were performed in triplicate and repeated independently at least three times.

The treatment duration and concentrations were determined based on preliminary dose-response and time-course analyses in both PC-3 and HPrEC.

### 2.4. Measurement of Glycolytic Metabolism

Glycolytic metabolism was assessed by measuring glucose consumption and lactate production in culture supernatants using commercially available colorimetric assay kits, according to the manufacturers’ protocols (cat. no. K606-100 for glucose and K627-100 for lactate, BioVision, Inc., Milpitas, CA, USA). Glucose utilization was calculated based on the reduction in glucose concentration relative to control media. Lactate production was normalized to cell number. Enzymatic activities of hexokinase (HK) and pyruvate dehydrogenase (PDH) were determined using an HK Colorimetric Assay Kit (cat. no. K789-100) and a PDH Activity Colorimetric Assay Kit (cat. no. K679-100), respectively (BioVision), and the results were expressed as percentages relative to control cells.

### 2.5. Intracellular ATP Measurement

Intracellular ATP levels were quantified using a luminescence-based ATP assay kit (cat. no. G9241, Promega Corporation, Madison, WI, USA). Following treatment, cells were lysed, and ATP content was measured according to the manufacturer’s instructions. Luminescence signals were recorded using a microplate reader and normalized to protein concentration.

### 2.6. Crystal Violet Biomass Assay

Cellular biomass was evaluated using crystal violet staining in both qualitative and quantitative formats. For qualitative analysis, cells were seeded in 6-well plates, treated as indicated, fixed with paraformaldehyde, and stained with crystal violet. Representative images were obtained to visualize treatment-dependent changes in cell biomass.

For quantitative analysis, cells were seeded in 96-well plates, and subjected to identical treatment conditions. After staining, excess dye was removed by washing, and bound crystal violet was solubilized using acetic acid. Absorbance was measured at 590 nm using a microplate reader, while biomass levels were expressed relative to untreated control cells.

### 2.7. Analysis of Apoptosis

Apoptotic cell populations were analyzed using the Muse™ Annexin V & Dead Cell Assay Kit (Merck KGaA, Darmstadt, Germany), according to the manufacturer’s protocol. Following treatment, cells were harvested, stained with Annexin V and viability dye, and analyzed using a Muse™ Cell Analyzer. Apoptotic cells were quantified as a percentage of the total cell population.

### 2.8. Western Blot Analysis

Cells were seeded at a density of 1 × 10^5^ cells per well in 6-well plates and allowed to attach overnight. Cells were then treated with GEN, BTN, or their combination (GEN/BTN) at the indicated concentrations for 48 h. Following treatment, whole-cell lysates were prepared using RIPA buffer (1× PBS, 1% NP-40, 0.5% sodium deoxycholate, 0.1% SDS, and 10 μg/mL phenylmethylsulfonyl fluoride). Protein concentrations were determined by bicinchoninic acid (BCA) assay (Thermo Fisher Scientific, Waltham, MA, USA).

Equal amounts of protein (40 μg) were separated on NuPAGE 4–12% Bis-Tris gels (Invitrogen, Waltham, MA, USA) and transferred onto polyvinylidene fluoride (PVDF) membranes (Cytiva, Marlborough, MA, USA). Membranes were incubated with primary antibodies against phosphor-AKT (Ser473; Cell Signaling Technology, Danvers, MA, USA #9271), total AKT (#9272), phosphor-ERK1/2 (Thr202/Tyr204; #4370), ERK1/2 (#4695), cleaved caspase-3 (#9664), cleaved poly(ADP-ribose) polymerase (PARP; #5625), hexokinase II (HK2 #2867), and pyruvate dehydrogenase (PDH; #3205) for 2 h at room temperature. All primary antibodies from Cell Signaling Technology were used at a dilution of 1:500. An anti-β-actin antibody (Sigma-Aldrich, A2228) was used as a loading control at a dilution of 1:1000. Membranes were subsequently incubated with horseradish peroxidase-conjugated secondary antibodies.

Immunoreactive bands were detected using an enhanced chemiluminescence (ECL) detection system (cat. no. W1001; Promega) and visualized on X-ray film. To confirm equal loading and enable comparative analysis, membranes were stripped and reprobed with antibodies against total AKT, total ERK, or β-actin, as appropriate. Densitometric analysis was performed using ImageJ software (version 1.0; National Institutes of Health, Bethesda, MD, USA), and protein expression levels were normalized to β-actin.

### 2.9. Statistical Analysis

All experiments were performed independently at least three times. Data are presented as the mean ± standard deviation (SD). Statistical analyses were conducted using one-way analysis of variance (ANOVA), followed by appropriate post hoc tests. A *p*-value < 0.05 was considered statistically significant.

## 3. Results

### 3.1. Effects of Genistein and Butein on Cell Viability in PC-3 and HPrEC Cells

**Figure 1:**
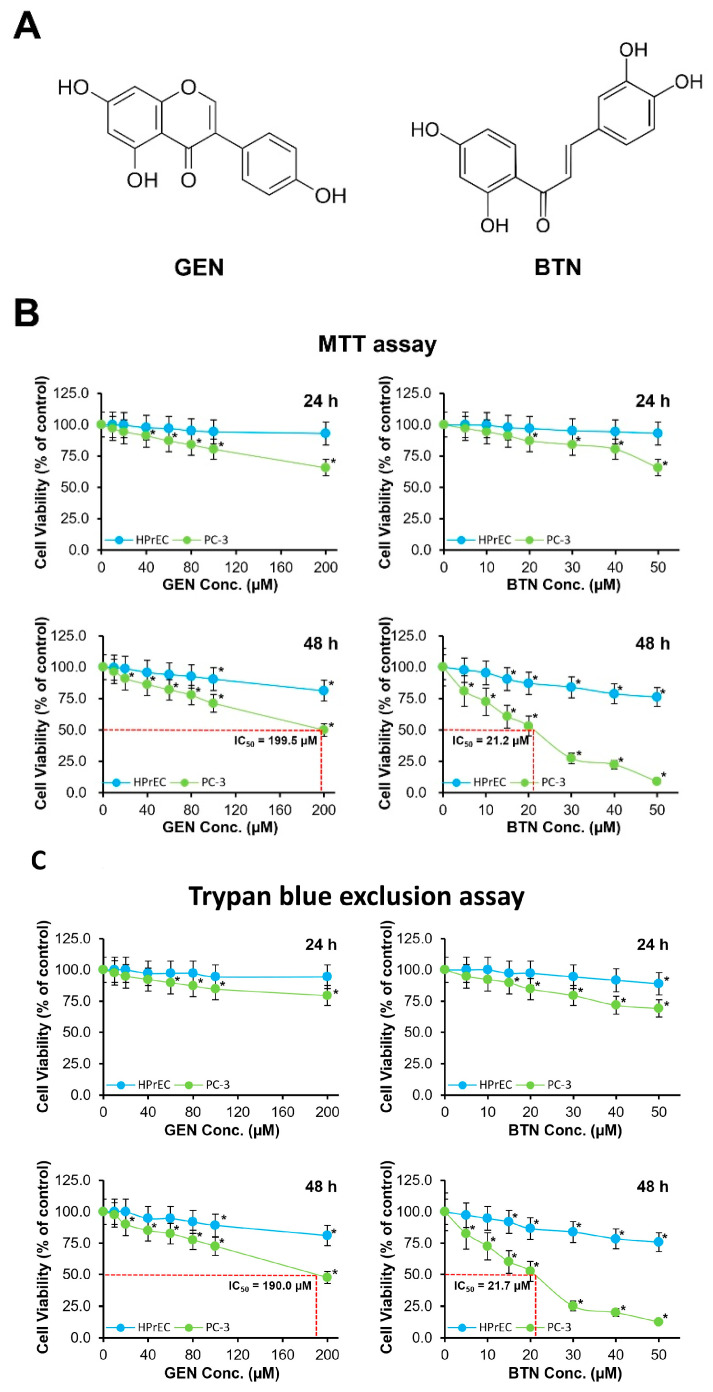
Genistein and butein reduce cell viability in prostate cancer cells. (**A**) Chemical structures of GEN and BTN. (**B**) Viability of PC-3 prostate cancer cells and HPrEC following exposure to increasing concentrations of GEN or BTN for 24 or 48 h, evaluated using the MTT assay. (**C**) Cell viability measured by means of trypan blue exclusion under identical treatment conditions. Results are normalized to untreated controls, and IC_50_ values were calculated from 48 h treatment data where indicated. Values represent the mean ± SD of at least three independent experiments; * *p* < 0.05 versus control. HPrEC: normal human prostate epithelial cells; GEN: genistein; BTN: butein; Conc.: concentration.

The effects of GEN and BTN on cell viability were evaluated in human prostate cancer PC-3 and HPrEC cells using MTT and trypan blue exclusion assays (Figure 1B,C). Figure 1A shows the chemical structures of GEN and BTN.

In the MTT assay, 24 h treatment with GEN resulted in minimal effects on HPrEC viability, which, up to 100 μM, remained above 95%, and at 200 μM, decreased slightly to 92.9% (Figure 1B, upper left). In contrast, PC-3 cells exhibited a concentration-dependent reduction in viability, decreasing at (100 and 200) μM to (80.4 and 65.6)%, respectively. After 48 h, GEN further reduced PC-3 viability at (100 and 200) μM to (71.2 and 49.9)%, respectively, while up to 100 μM, HPrEC viability remained above 90% (Figure 1B, lower left). The calculated IC_50_ value of GEN in PC-3 cells at 48 h was approximately 199.5 μM.

BTN treatment produced a more pronounced cytotoxic effect in PC-3 cells (Figure 1B, right panels). At 24 h, PC-3 viability at (30 and 50) μM decreased to (68.2 and 55.8)%, whereas HPrEC viability remained above (89 and 81.5)%, respectively. After 48 h, BTN markedly reduced PC-3 viability at (30 and 50) μM to (27.3 and 8.9)%, while HPrEC cells retained (84.0 and 76.1)% viability, respectively. The estimated IC_50_ value of BTN in PC-3 cells at 48 h was 21.2 μM.

Trypan blue exclusion assays yielded results consistent with the MTT data (Figure 1C). After 48 h, GEN reduced PC-3 cell viability at 200 μM to 47.5%, whereas BTN reduced viability at 50 μM to 12.5%, while under both treatment conditions, HPrEC viability remained above 80%. These findings confirm the selective cytotoxic effects of GEN and BTN toward PC-3 cells.

Comparative analyses at 24 and 48 h revealed that at 24 h, treatment-induced changes in viability and metabolic parameters were limited, but at 48 h, became clearly distinguishable and reproducible, particularly in PC-3 cells. Therefore, a 48 h treatment period was selected for subsequent viability and mechanistic experiments. The concentrations used for single and combination treatments were chosen to minimize nonspecific cytotoxicity in normal cells. At the selected concentrations of 100 μM GEN and 15 μM BTN, HPrEC viability consistently remained above 90% across MTT, trypan blue exclusion, and crystal violet assays, indicating minimal toxicity, while producing pronounced inhibitory effects in PC-3 cells.

### 3.2. GEN/BTN Co-Treatment Suppresses Glycolytic Metabolism in PC-3 Cells

**Figure 2:**
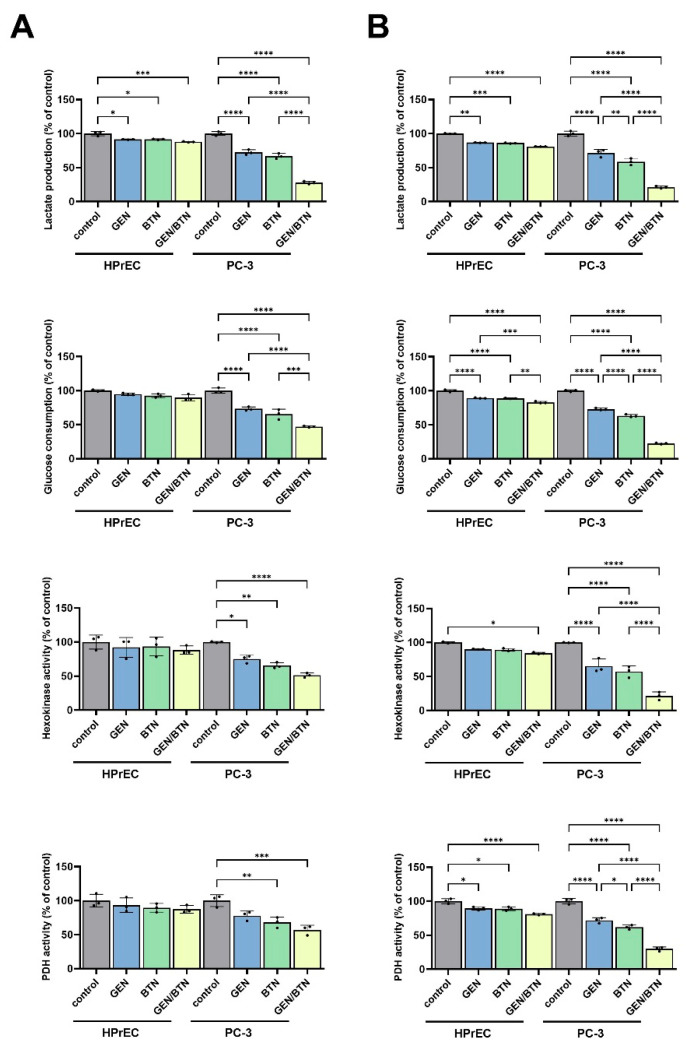
Combined genistein and butein treatment impairs glycolytic metabolism in PC-3 cells. HPrEC and PC-3 cells were treated with GEN, BTN, or GEN/BTN for 48 h. (**A**,**B**) Extracellular lactate production and glucose consumption were quantified and normalized to control levels. Enzymatic activities of hexokinase (HK) and pyruvate dehydrogenase (PDH) were assessed following treatment. GEN/BTN co-treatment produced a pronounced suppression of glycolytic activity in PC-3 cells, whereas metabolic parameters in HPrEC cells remained largely unchanged. Data are shown as the mean ± SD (n = 3). Statistical significance is indicated by asterisks (* *p* < 0.05; ** *p* < 0.01; *** *p* < 0.001; **** *p* < 0.0001). HPrEC: normal human prostate epithelial cells; GEN: genistein; BTN: butein.

To investigate whether GEN–BTN-induced cytotoxicity was associated with metabolic alterations, lactate production, glucose consumption, and the activities of key metabolic enzymes were examined in PC-3 and HPrEC cells after 24 and 48 h of treatment (Figure 2).

Figure 2A (24 h) shows that following GEN (100 μM) and BTN (15 μM) treatment, lactate production in PC-3 cells was reduced to (72.5 and 66.8)% of control levels, respectively, whereas GEN/BTN co-treatment markedly reduced lactate production to 27.7%. In contrast, under all treatment conditions, HPrEC cells exhibited only modest reductions, maintaining lactate production above 87.9%. After 48 h (Figure 2B), lactate production in PC-3 cells with GEN, BTN, and GEN/BTN co-treatment was further decreased to (71.4, 58.6, and 21.2)%, respectively, while HPrEC cells retained more than 80.9% of control levels.

Glucose consumption displayed a similar pattern. At 24 h (Figure 2A) after GEN and BTN treatment, glucose uptake in PC-3 cells decreased to (73.3 and 65.4)%, respectively, and was further reduced by GEN/BTN co-treatment to 47.1%. After 48 h (Figure 2B), glucose consumption in PC-3 cells for GEN, BTN, and GEN/BTN declined to (72.9, 63.2, and 22.6)%, respectively; in contrast, HPrEC cells maintained glucose consumption above 83.0%, even after co-treatment, indicating relative metabolic resistance.

Consistent with these findings, enzymatic activities of hexokinase (HK) and pyruvate dehydrogenase (PDH) were significantly inhibited in PC-3 cells (Figure 2A,B). At 24 h (Figure 2A), HK activity in PC-3 cells for GEN, BTN, and GEN/BTN treatment decreased to (75.0, 65.5, and 51.3)%, whereas PDH activity declined to (78.0, 68.2, and 57.0)%, respectively. At 48 h, these inhibitory effects were more pronounced (Figure 2B), with HK activity for GEN, BTN, and GEN/BTN reduced to (65.3, 57.2, and 21.5)%, while PDH activity decreased to (71.9, 61.7, and 29.7)%, respectively.

Notably, in HPrEC cells, HK and PDH activities under all treatment conditions at both time points remained above 80% of control levels (Figure 2A,B).

### 3.3. GEN/BTN Co-Treatment Induces ATP Depletion and Suppresses Cellular Biomass in PC-3 Cells

**Figure 3:**
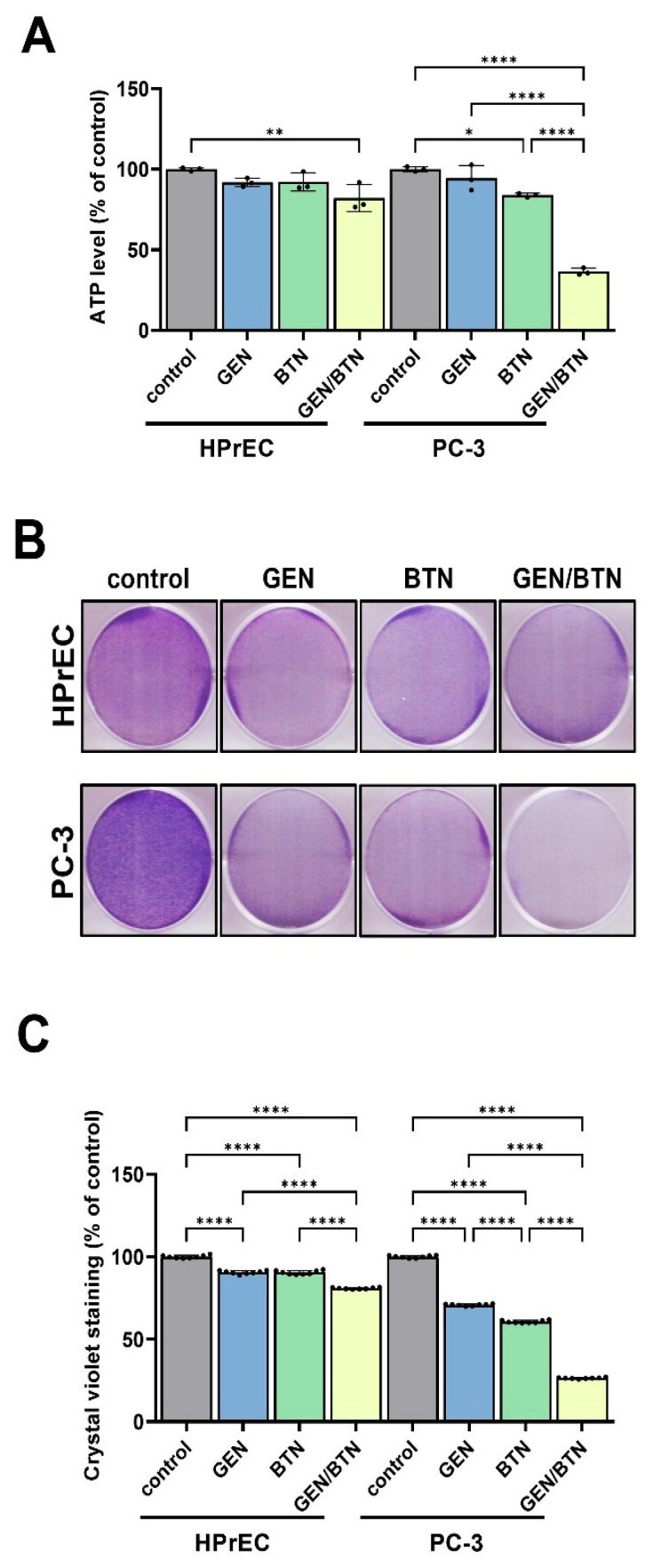
Genistein–butein co-treatment decreases ATP levels and clonogenic survival in PC-3 cells. (**A**) Intracellular ATP content was determined after 48 h exposure to GEN, BTN, or GEN/BTN, and expressed relative to control cells (n = 3 independent experiments). (**B**) Crystal violet staining images illustrating treatment-dependent changes in cell survival. (**C**) Quantitative analysis of crystal violet staining measured at 590 nm. Each dot represents an individual replicate, and data are presented as mean ± SD (total n = 8 pooled from at least three independent experiments). Following GEN/BTN co-treatment in PC-3 cells, a significant reduction in ATP production and cell survival was observed, while normal HPrEC cells showed minimal changes. Results represent the mean ± SD from three independent experiments. Statistical significance is indicated by asterisks (* *p* < 0.05; ** *p* < 0.01; **** *p* < 0.0001). HPrEC: normal human prostate epithelial cells; GEN: genistein; BTN: butein.

To determine whether GEN/BTN-induced metabolic disruption translated into impaired cellular energy status and growth, intracellular ATP levels and cellular biomass were evaluated after 48 h of treatment (Figure 3).

Figure 3A shows that GEN and BTN treatment moderately reduced ATP levels in PC-3 cells to (73.4 and 64.7)% of control values, respectively. Notably, GEN/BTN co-treatment resulted in a marked depletion of intracellular ATP, reducing ATP levels to 25.1% of control. In contrast, ATP levels in HPrEC cells remained largely preserved, for GEN, BTN, and GEN/BTN measuring (91.2, 91.5, and 80.9)%, respectively, indicating selective energy depletion in prostate cancer cells.

The effects of GEN/BTN co-treatment on cell growth were further assessed using crystal violet staining (Figure 3B,C). Representative images from 6-well plates (Figure 3B) demonstrated a pronounced reduction in staining intensity in PC-3 cells following GEN/BTN co-treatment compared with single-agent treatments, whereas HPrEC cells exhibited only modest changes.

Quantitative analysis using crystal violet solubilization in 96-well plates (Figure 3C) confirmed these observations. In PC-3 cells, GEN and BTN treatment reduced cellular biomass to (70.8 and 60.6)% of control levels, respectively, whereas GEN/BTN co-treatment dramatically suppressed biomass to 26.4%. In contrast, for GEN, BTN, and GEN/BTN treatment, HPrEC cells retained (90.5, 90.4, and 80.8)% of control biomass levels, respectively. Representative crystal violet-stained 96-well plate images are provides in the Supplementary Materials (Supplementary Figure S1).

### 3.4. GEN/BTN Co-Treatment Suppresses Survival Signaling and Induces Apoptosis in PC-3 Cells

**Figure 4:**
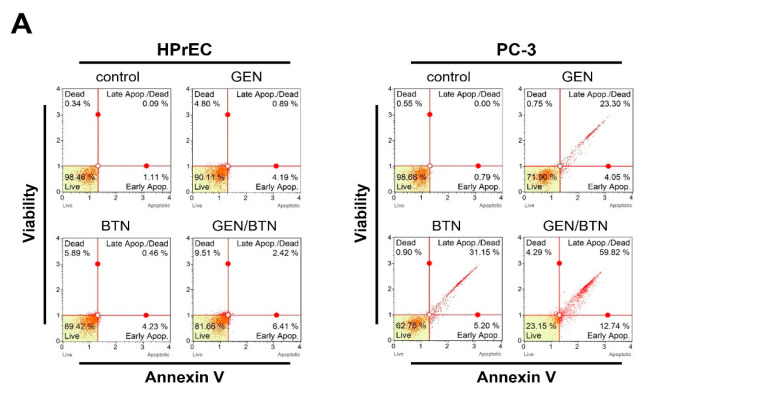

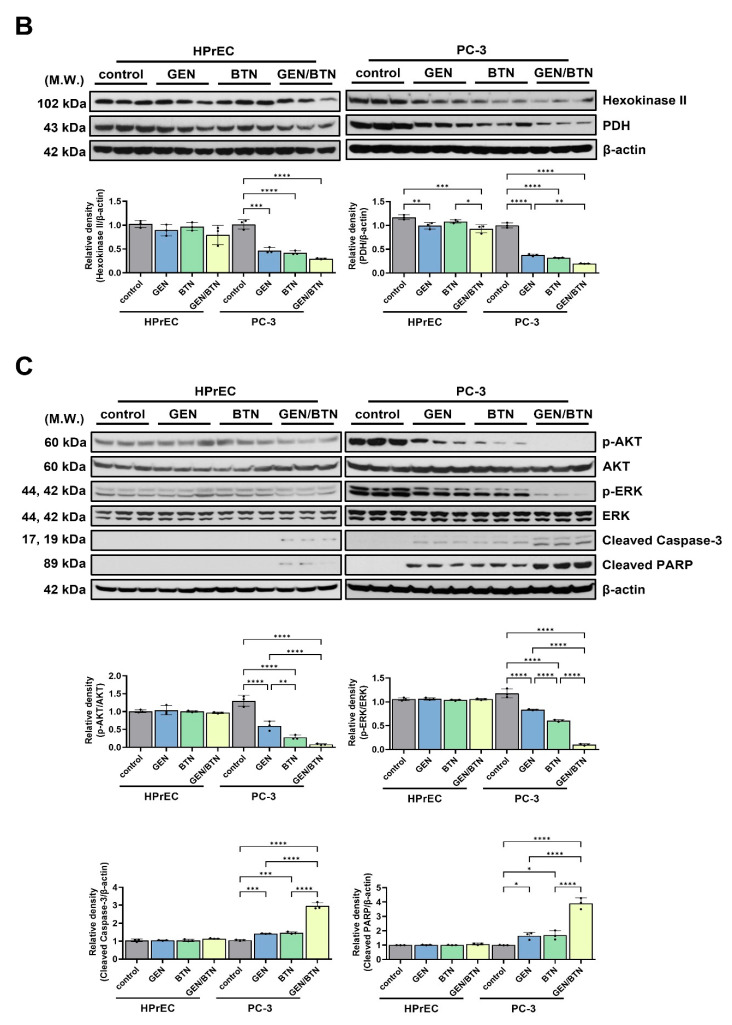
Genistein–butein co-treatment promotes apoptosis and suppresses AKT/ERK signaling in PC-3 cells. (**A**) Apoptotic cell populations were evaluated using Annexin V and viability staining after 48 h treatment. Representative dot plots are shown for HPrEC and PC-3 cells. (**B**) Expression levels of hexokinase II and PDH were analyzed by means of Western blotting, with β-actin serving as a loading control. Corresponding densitometric analyses are presented. (**C**) Phosphorylation status of AKT and ERK, along with levels of cleaved caspase-3 and cleaved PARP, was examined by Western blot analysis. GEN/BTN co-treatment selectively inhibited survival signaling and enhanced apoptotic marker cleavage in PC-3 cells. Data are presented as the mean ± SD. Statistical significance is indicated. Statistical significance is indicated by asterisks (* *p* < 0.05; ** *p* < 0.01; *** *p* < 0.001; **** *p* < 0.0001). HPrEC: normal human prostate epithelial cells; GEN: genistein; BTN: butein; PDH: pyruvate dehydrogenase.

To elucidate the molecular mechanisms underlying GEN/BTN-mediated growth inhibition, apoptotic induction and survival signaling pathways were examined in PC-3 and HPrEC cells after 48 h of treatment (Figure 4).

Flow cytometric analysis in PC-3 cells using Annexin V staining demonstrated, following GEN/BTN co-treatment, a pronounced induction of apoptosis (Figure 4A). In PC-3 cells, GEN or BTN alone moderately increased apoptotic populations, whereas co-treatment markedly elevated both early and late apoptotic fractions, accompanied by a substantial reduction in viable cell populations. In contrast, HPrEC cells exhibited only minimal changes in apoptotic distribution across all treatment conditions, indicating selective apoptotic induction in prostate cancer cells.

Given the observed metabolic suppression, expression levels of key glycolytic enzymes were assessed by Western blot analysis (Figure 4B). In PC-3 cells, densitometric analysis revealed that hexokinase II protein levels for GEN, BTN, and GEN/BTN treatment were reduced to (0.46, 0.411, and 0.29)-fold, respectively, relative to control. Similarly, in PC-3 cells, pyruvate dehydrogenase (PDH) expression for GEN, BTN, and GEN/BTN treatment was markedly decreased to (0.377, 0.319, and 0.198)-fold, respectively; in contrast, HPrEC cells showed only modest reductions in HK2 and PDH expression, remaining close to control levels.

To further investigate survival signaling pathways, AKT and ERK phosphorylation status and apoptotic cleavage markers were analyzed (Figure 4C). In PC-3 cells, GEN/BTN.

Co-treatment strongly suppressed pro-survival signaling, with p-AKT and p-ERK levels reduced to (0.062 and 0.085)-fold of control values, respectively, while total AKT and ERK levels remained unchanged. Concomitantly, apoptotic execution markers were robustly induced, as evidenced by a (2.795 and 3.88)-fold increase in cleaved caspase-3 and in cleaved PARP expression, respectively. In contrast, HPrEC cells following treatment displayed minimal alterations in AKT/ERK phosphorylation and apoptotic cleavage.

## 4. Discussion

Metabolic reprogramming is a defining feature of cancer cells, allowing malignant populations to adapt to fluctuating nutrient availability and sustain uncontrolled proliferation. In prostate cancer, enhanced reliance on glycolysis and altered mitochondrial function have been associated with disease progression and therapeutic resistance, making metabolic pathways attractive targets for intervention [16,17,18]. The present study demonstrates that combined treatment with GEN and BTN selectively disrupts metabolic homeostasis in PC-3 prostate cancer cells, leading to energy depletion, suppression of survival signaling, and activation of apoptotic pathways, while largely sparing normal prostate epithelial cells.

Initial viability analyses revealed that GEN and BTN exerted concentration- and time-dependent effects on PC-3 cells, with substantially greater sensitivity compared to HPrEC cells. While after 24 h of treatment, moderate reductions in cancer cell viability were observed, at 48 h, pronounced cytotoxicity, metabolic suppression, and apoptotic activation became evident. Accordingly, the 48 h time point was selected as the primary mechanistic window, as it allowed sufficient accumulation of metabolic stress to translate into ATP depletion, signaling inhibition, and execution-phase apoptosis, without inducing secondary necrotic effects that can confound mechanistic interpretation [19,20]. This temporal selection ensured that downstream molecular alterations reflected regulated metabolic failure, rather than nonspecific cellular damage.

Dose selection for combination studies was guided by stringent biological criteria emphasizing cancer selectivity. Based on dose–response profiling, GEN and BTN at (100 and 15) μM, respectively, were identified as concentrations in PC-3 cells that produced robust anticancer effects, while in HPrEC cells maintaining ≥ 90% viability. This approach minimized nonspecific cytotoxicity and preserved the physiological integrity of normal epithelial cells, thereby enabling the interrogation of cancer-selective metabolic vulnerability, rather than generalized chemical stress. Such an experimental design is particularly relevant in nutritional and metabolic oncology, where therapeutic relevance depends on the selective targeting of malignant cells [21,22].

Metabolic analyses demonstrated that GEN/BTN co-treatment markedly suppressed glycolytic output in PC-3 cells, as evidenced by significant reductions in glucose consumption and lactate production. These effects were substantially more pronounced than those observed with either compound alone, while being largely absent in HPrEC cells. Cancer cells frequently depend on sustained glycolytic flux to support biosynthesis and redox balance, even in the presence of oxygen, a phenomenon commonly referred to as the Warburg effect [23,24]. The observed suppression of glucose utilization and lactate generation suggests that GEN/BTN interferes with both substrate uptake and downstream glycolytic processing, thereby limiting metabolic flexibility in prostate cancer cells. Collectively, these results demonstrate that GEN/BTN co-treatment selectively disrupts glycolytic metabolism in PC-3 cells by suppressing glucose utilization, lactate production, and key metabolic enzyme activities, while largely sparing normal prostate epithelial cells.

At the enzymatic level, combined treatment in PC-3 cells significantly reduced both the activity and expression of hexokinase II and PDH. Hexokinase II is a key regulator of glycolytic entry and is often overexpressed in aggressive cancers, where it also contributes to apoptosis resistance through mitochondrial association [25,26,27]. PDH serves as a metabolic gatekeeper linking glycolysis to mitochondrial oxidative metabolism, while its inhibition further restricts cellular energy production [28,29]. The coordinated suppression of hexokinase II and PDH thus represents a dual blockade of glucose metabolism, effectively uncoupling glycolysis from mitochondrial ATP generation.

Consistent with enzymatic inhibition, following GEN/BTN co-treatment, intracellular ATP levels in PC-3 cells were profoundly reduced, whereas in HPrEC cells, they remained largely preserved. Energy depletion represents a critical stress signal in cancer cells, which typically operate near maximal bioenergetic capacity, and possess limited ability to compensate for metabolic disruption [30,31]. Collectively, these results demonstrate that in PC-3 cells, GEN/BTN co-treatment induces profound ATP depletion and significantly suppresses cellular biomass, while in normal prostate epithelial cells exerting relatively limited effects. These findings indicate that metabolic energy depletion contributes directly to the growth-inhibitory effects of GEN/BTN co-treatment in prostate cancer cells.

Beyond metabolic perturbation, GEN/BTN co-treatment strongly suppressed pro-survival signaling pathways. In PC-3 cells, phosphorylation of AKT and ERK was markedly reduced, while total protein levels remained unchanged, indicating inhibition of pathway activation, rather than protein expression. AKT and ERK signaling are central regulators of metabolic adaptation, cell growth, and survival, and their activation is frequently associated with resistance to metabolic stress and anticancer therapies [32,33,34]. Suppression of these pathways likely amplifies metabolic vulnerability by limiting compensatory survival responses.

Concomitant with signaling inhibition, robust activation of apoptotic pathways was observed in PC-3 cells. Flow cytometric analysis demonstrated a marked increase in apoptotic populations following GEN/BTN co-treatment, while Western blot analysis confirmed significant induction of cleaved caspase-3 and cleaved PARP [35,36]. These findings indicate engagement of execution-phase apoptosis downstream of metabolic and signaling disruption [37,38,39]. In contrast, HPrEC cells exhibited minimal changes in apoptotic markers, underscoring the cancer-selective nature of GEN/BTN-induced cell death. Collectively, these results demonstrate that GEN/BTN co-treatment selectively suppresses AKT/ERK-mediated survival signaling while activating apoptotic pathways in PC-3 cells, providing a mechanistic link between metabolic dysfunction and apoptotic cell death.

**Figure 5:**
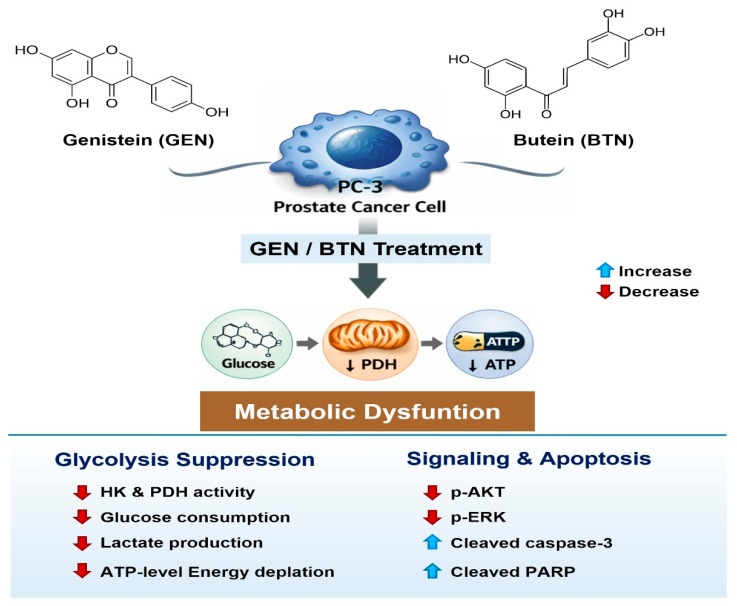
Schematic overview of the molecular mechanisms underlying genistein-butein-induced metabolic dysfunction and apoptosis in PC-3 cells. The schematic illustrates that combined treatment with GEN and BTN disrupts glycolytic metabolism by reducing hexokinase and pyruvate dehydrogenase activity, glucose utilization, lactate production, and intracellular ATP levels. In parallel, suppression of AKT and ERK signaling facilitates the activation of apoptotic pathways, as evidenced by the increased cleavage of caspase-3 and PARP. These coordinated effects ultimately contribute to the reduced viability of PC-3 prostate cancer cells. HK: hexokinase; PDH: pyruvate dehydrogenase; GEN: genistein; BTN: butein.

Importantly, the integration of viability, metabolic, enzymatic, and signaling data supports a unified mechanistic model in which GEN/BTN co-treatment exploits metabolic dependencies that are unique to prostate cancer cells. Figure 5 illustrates that combined treatment disrupts glycolytic metabolism by suppressing hexokinase and PDH activity, leading to reduced glucose consumption, diminished lactate production, and ATP depletion [40,41,42,43,44,45]. This metabolic dysfunction is accompanied by the inhibition of AKT and ERK signaling, lowering the apoptotic threshold, and culminating in caspase-mediated cell death [42,43,44]. Normal prostate epithelial cells, which rely less heavily on glycolytic flux and retain greater metabolic plasticity, are comparatively resistant to these perturbations [45,46,47,48].

Collectively, these findings highlight the potential of phytochemical combinations to selectively target cancer metabolism, while minimizing toxicity to normal tissues [37,38,39]. By explicitly defining treatment duration and concentration based on cancer selectivity and mechanistic clarity, this study provides a robust framework to evaluate metabolism-targeted strategies in prostate cancer [46,47,48]. The results support further investigation of GEN/BTN co-treatment as a nutritionally relevant approach to exploit cancer-selective metabolic vulnerability [49,50].

Several limitations of the present study should be acknowledged. First, the mechanistic effects of genistein and butein co-treatment were evaluated in a single androgen-independent prostate cancer cell line (PC-3), and therefore the generalizability of these findings to other prostate cancer subtypes remains to be established. Second, while the present data clearly demonstrate suppression of glycolytic metabolism accompanied by inhibition of AKT/ERK signaling and induction of apoptotic cleavage, direct analyses of mitochondrial respiration and metabolic flux would provide a more comprehensive understanding of the metabolic reprogramming induced by the combination treatment [46,47,48]. Finally, in vivo validation using prostate cancer xenograft or orthotopic models will be required to further substantiate the biological and translational relevance of these findings [49,50].

Despite these limitations, the present study provides compelling mechanistic evidence that combined genistein and butein treatment exerts a cooperative anticancer effect in PC-3 prostate cancer cells by coordinately suppressing glycolytic metabolism and survival signaling pathways while activating apoptotic execution mechanisms [35,36,37,40,41,42,43,44,45]. The simultaneous downregulation of hexokinase II-, PDH-, and AKT/ERK-associated signaling, together with enhanced caspase-3 and PARP cleavage, highlights metabolic vulnerability as a key target of this phytochemical combination [45,47,48,49,50]. Collectively, these findings provide a molecular basis for the rational design of polyphenol-based co-treatment strategies aimed at disrupting metabolic homeostasis in aggressive prostate cancer cells [49,50].

## 5. Conclusions

This study demonstrates that combined treatment with genistein and butein selectively suppresses prostate cancer cell growth by targeting metabolic dependencies preferentially exploited by malignant cells. GEN/BTN co-treatment disrupts glycolytic metabolism through the coordinated inhibition of glucose utilization, lactate production, and the activities of hexokinase and pyruvate dehydrogenase, resulting in pronounced ATP depletion in PC-3 cells. This metabolic stress is accompanied by the suppression of AKT and ERK signaling and the activation of caspase-dependent apoptotic pathways, culminating in reduced cell viability and cellular biomass.

Importantly, these effects were achieved at concentrations that preserved high viability in normal prostate epithelial cells, indicating minimal nonspecific cytotoxicity while highlighting a cancer-selective therapeutic window. The deliberate selection of treatment duration and concentration allowed mechanistic dissection of metabolic vulnerability without confounding toxic effects. Collectively, these findings provide mechanistic insight into the synergistic anticancer actions of dietary polyphenol combinations and support further investigation of metabolism-targeted strategies as complementary approaches in prostate cancer research.

## Data Availability

The original contributions presented in the study are included in the article; further inquiries should be directed to the corresponding author.

## Supplementary Materials

The following supporting information can be downloaded at https://www.mdpi.com/article/10.3390/cimb48030258/s1.

## Abbreviations

The following abbreviations are used in this manuscript:

AKT	protein kinase B
ATP	adenosine triphosphate
BTN	butein
CV	crystal violet
DMEM	Dulbecco’s modified Eagle medium
DMSO	dimethyl sulfoxide
ERK	extracellular signal-regulated kinase
GEN	genistein
HK	hexokinase
HPrEC	human prostate epithelial cells
MTT	3-(4,5-dimethylthiazol-2-yl)-2,5-diphenyltetrazolium bromide
PARP	poly(ADP-ribose) polymerase
PC-3	human prostate cancer cell line
PDH	pyruvate dehydrogenase
PI	propidium iodide
ROS	reactive oxygen species
