# Supplementary material for: Genistein–Butein Co-Treatment Suppresses Glycolytic Metabolism and Induces Apoptotic Signaling in PC-3 Prostate Cancer Cells
Source: Curr Issues Mol Biol. 2026 Feb 27;48(3):258. doi: 10.3390/cimb48030258 (PMC13025362; doi:10.3390/cimb48030258)
Supplement: Supplementary file 1 [file cimb-48-00258-s001.zip › cimb-4159681-supplementary.pdf]

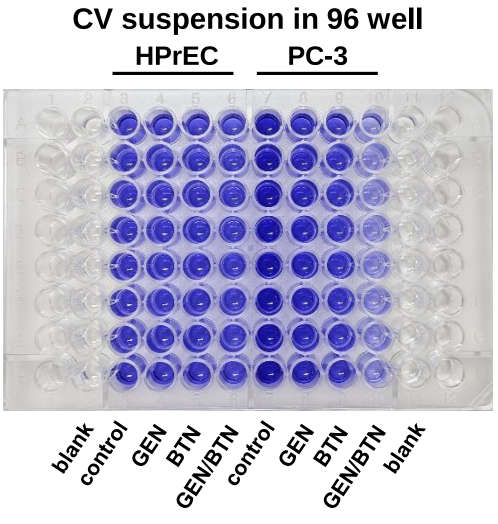

Figure S1: Representative multiwell crystal violet staining images of HPrEC and PC-3 cells following GEN, BTN, or GEN/BTN treatment.
